# Review of Current Healthcare Waste Management Methods and Their Effect on Global Health

**DOI:** 10.3390/healthcare9030284

**Published:** 2021-03-05

**Authors:** Christina Kenny, Anushree Priyadarshini

**Affiliations:** 1 College of Business, Technological University Dublin, 2 Dublin, Ireland; d18125471@mytudublin.ie; 2 Environment Sustainability and Health Institute, Technological University Dublin, 7 Dublin, Ireland

**Keywords:** waste disposal, medicine, global warming, public health, environment, incineration, landfilling, irradiation

## Abstract

Healthcare is a rapidly growing industry as medical treatments become more sophisticated, more in demand due to increasing incidence of chronic disease and more widely available worldwide. This booming industry is also creating more waste than ever before and, as such, there is a growing need to treat and dispose of this waste. Healthcare waste (HCW) disposal includes a multitude of disposal methods, including incineration, landfilling and chemical treatments. These rudimentary methods and their growing use present their own problems that negatively impact both the environment and, in turn, damage public health, thus contributing to a global healthcare crisis. The aim of this review was to examine the current HCW disposal methods in place and the harmful effects they have on the environment and on public health. The findings accumulated in this review demonstrate a heavy reliance on basic, low tech HCW disposal techniques and uncovered the negative impacts of these methods. There is a notable lack of employment of “greener” HCW disposal methods on a largescale due to cost, access and feasibility. Despite innovations in HCW disposal, there is no scalable, global green solution at present. Further, the review highlights that global health consequences of HCW disposal methods often differ depending on how developed the country is.

## 1. Introduction

Healthcare has become one of the fastest growing global industries in recent years [1]. Worldwide health spending is higher than ever and continues to rise every year. In 2017, global health spending was estimated at USD 7.8 trillion worldwide and was around 10% of the world gross domestic product (GDP) [2]. This increase in spending is due to expanding population numbers and a greater need for healthcare interventions. The global population is increasing and is expected to continue to do so, with it estimated to be around 8.6 billion by 2030 and 9.8 billion by 2050 [3]. Consequently, there would be a need for more healthcare interventions and treatments as the population both expands and lives longer resulting in generation of more healthcare waste (HCW) than ever before.

With aging population, while chronic disease spending is continuing to grow, so too is the cost of having multiple morbid conditions (MMCs). MMCs lead to elevated use of use of primary care and specialist care services as well as increased medication use and elevated hospital admissions [4]. Rapoport et al. [5]. demonstrated that the use of clinician appointments increases by up to 51% with each additional chronic disease. As age increases so too does the likelihood of suffering with a chronic disease [6]. Having one chronic disease also makes it more likely that someone will suffer numerous associated comorbidities leading to further, complicated chronic disease management. This growing epidemic of chronic disease has meant that the HCW disposal practices are being utilised extensively and the HCW disposal industry is growing. Alongside the ongoing growth of chronic disease, both developed and developing countries around the world are carrying out improved contagion practices in order to handle pandemics such as Ebola and COVID-19 and as a result more potentially dangerous medical waste is being produced [7,8]. Infectious diseases are also increasing globally adding to the generation of more waste [9]. At present, around 1.47 billion tonnes of solid waste are produced each year globally and this figure has increased year on year [10]. Of these 1.47 billion tonnes, 5.9 million tonnes are estimated to be a result of HCW [11]. Yet, owing to the sensitive nature and rigorous legislative requirements, only limited studies have explored the HCW streams in detail to put forward optimum methods for their treatment [12].

European Union legislation separates HCW into the following three categories of materials: (i) any HCW that poses a risk of infection; (ii) HCW that is a chemical hazard; and (iii) medicines and medicinally contaminated waste containing a pharmaceutically active agent [13]. Almost 85% of waste generated by healthcare activity is considered ‘non-hazardous waste’ and the remaining 15% is labelled as ‘hazardous’ [14]. While non-hazardous waste may not sound dangerous, the means of safely disposing of this waste can have damaging effects on the environment. The ‘hazardous’ waste can be infectious, toxic or even radioactive [14] and hence comes with a host of disposal and non-disposal issues. As such, not all HCW is created equally and different categories of waste require different disposal methods. Globally, the volume of HCW generated (per kg/bed/day) ranges enormously depending on the region and country (Table 1).

This increasing volume of HCW means a surge in use of HCW disposal practices such as landfilling, composting and the most preferred method: incineration [29] as well as other routes such as source reduction and reprocessing and recycling. HCW disposal practices have been associated with a wide range of problems. These problems, while potentially very dangerous for the environment, have an even greater, more widespread effect on people [41]. When proper disposal techniques are carried out correctly, such as incineration, there is risk of emission of pollutants like acid gases, oxides of nitrogen, metals, particulate matter and sulphur [42]. These pollutants have had notable negative effects on human health effecting the respiratory system, the endocrine system and have led to higher incidences of chronic diseases and cancers [43]. However, a range of factors like HCW type, classification, mechanism of segregation and waste management techniques impact the emissions from incineration of HCW [44,45]. Thus, it is essential to ensure that incinerators are operated as per advanced technological and legislation (such as European Community (EC) guidelines [46] to have clean and safe processes [47]. While, when HCW is not disposed of in the correct or proper manner due to a wide range of potential factors, such as lack of facilities or equipment, lack of education and training or a lack of regulation, it has additional disastrous effects on the environment and in turn, on human health [48]. Additionally, waste disposal within healthcare does not only concern hospitals and healthcare settings such as pharmacies, it also involves the patient as a key contributor. Bashaar et al. [49] have demonstrated that many adults dispose of unused or expired medications in unsafe and unofficial manners, leading to unsafe pharmaceutical compounds ending up in the environment and then eventually to the general public which has health repercussions. 

At present, every method of HCW disposal comes with disadvantages that not only pose a threat to the environment but also a threat to human health and wellbeing often via the environment. Efforts have been made in recent years to improve this counterproductivity in which HCW could be potentially harmful to human life. These efforts have led to a push for “greener” and “safer” means of HCW disposal such as autoclaving, microwaving and steam augor but even green methods come with a range of potential challenges as many are not suitable for large volumes of waste or are not widely available [50], moreover these methods tend to be supportive to conventional techniques than being their replacement. Such as a steam autoclave can sterilize bacteria in clinical solid waste but cannot be considered as an alternative technology to incineration due to the re-growth risk of the bacteria [51]. They have also led to further education for healthcare professionals and personnel on waste management and “green hospital” initiatives. Although attempts have been made to improve the process of HCW disposal, serious problems and safety issues related to public health still very much exist [52]. The growing global population, the increase in lifespan, and the global crisis in chronic disease mean not only is more HCW being produced than ever before but there is an even greater need to better manage it. Current methods of HCW disposal are limited in managing the global HCW problem. In order for substantial progress to be made on this, a more in-depth understanding of why this issue exists and the barriers to the design or innovation on improved HCW disposal technologies must first be explored. The true scope of just how damaging HCW disposal methods can be is not widely understood. It still remains an immense problem with disastrous global consequences to both health and well-being not to mention the damaging environmental effects. It is therefore critical for this problem to be better understood in order for global change to occur. Thus, this review aims to examine the depth of relationship between current disposal methods and how these negatively impact the global public health.

## 2. Methodology

The articles included in this review were obtained using multiple databases including Google Scholar, PubMed, Science Direct and Scopus. The main search terms used included: healthcare waste management, medical waste management, healthcare and environment, healthcare pollution, medicine and environment, medicine and pollution, healthcare greenhouse gases and greenhouses gases healthcare waste, etc. Eligibility criteria included papers that examined either the impacts of healthcare waste or practices on the environment or the link between the negative impact of healthcare waste on the environment and in turn, on public health. Only papers published within the last 20 years were included and reviewed and grey literature was not included in the search. All relevant papers available in English were reviewed. The initial screening process involved screening titles and abstracts and from there identifying suitability. All suitable articles were then read in full. Articles used in the review were then divided into relevant sections based on the HCW methods included or described or other relevant sections of the paper. Once primary papers were identified further, more specific searches on the same databases were carried out using more targeted terms such as: landfilling healthcare waste, pyrolysis of healthcare waste, etc.

A separate search was carried out for literature and documents produced by government bodies, NGOs (non-governmental organisations) and IGOs (Intergovernmental organisations) such as the United Nations, World Health Organisation, European Commission, etc. This was done by searching the websites of the organisations used directly for the necessary content.

## 3. Sources of HCW

**Figure 1:**
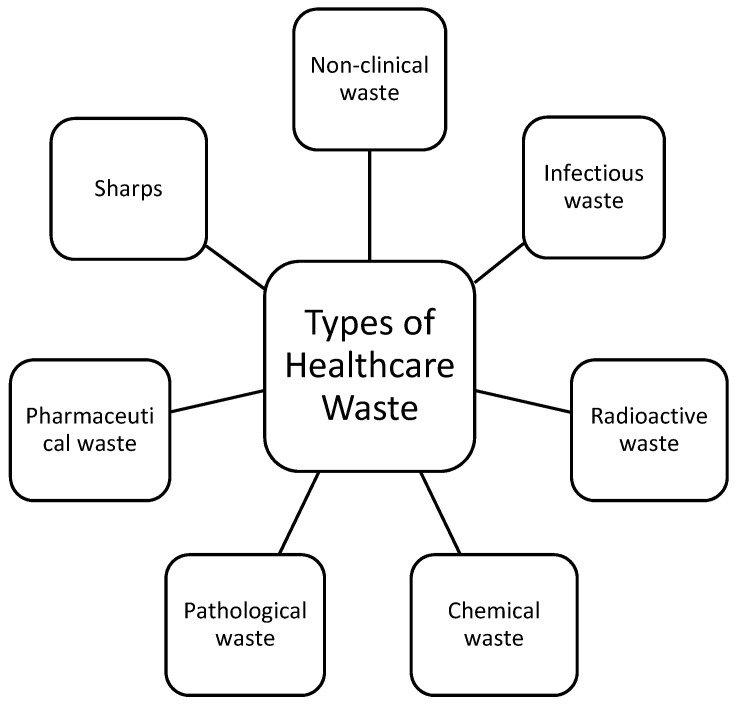
Types of HCW generated.

HCW waste exists in a wide range of forms with varying degrees of complexity and toxicity (Figure 1). It is separated into general or non-clinical waste and clinical waste. General waste is considered non-hazardous material that poses no potential danger and as such does not require highly specialised methods of disposal [53]. While clinical waste can pose a number of dangers, can be of varying degrees of toxicity depending on the nature of the waste and as such requires highly specialised handling and treatment. Most clinical waste comes from hospitals and clinics providing acute services such as Operating Theatres, Maternity services, Accident & Emergency, Mortuary, Intensive Care, Isolation Wards, Pharmacy, Pathology Laboratories and other research facilities and laboratories [34]. Clinical waste, to a lesser extent also comes from ambulatory services, public health laboratories, blood donation centres, dental surgeries, veterinary surgeons, vaccination clinics and hospitals, clinics and nursing homes providing community care, care of the elderly and mental health services [54,55].

## 4. Currently Utilised Methods of HCW Treatment and Disposal

There are numerous methods of HCW treatment and disposal available globally, despite this, the most widely utilised method is still incineration with landfilling following closely behind [56]. Various irradiation methods such as microwaving and various methods of thermal treatment such as autoclaving and steam treatment are much more environmentally friendly than the most commonly employed methods, but they are not suitable for mass, large scale treatment of waste and generally are not even available in many countries globally [55]. While greener methods technically do exist, they are unsuitable and unrealistic for numerous reasons including their lack of ability to treat large volumes, their need to exclude volatile materials and the steep, expensive price tag that comes along with them. Some of the greener methods also include prior steps before use and as such only carry out partial waste disposal [57]. Zimmermann [58] also highlight that the use of greener techniques such as microwave must be considered in the context of other treatment options.

**Figure 2:**
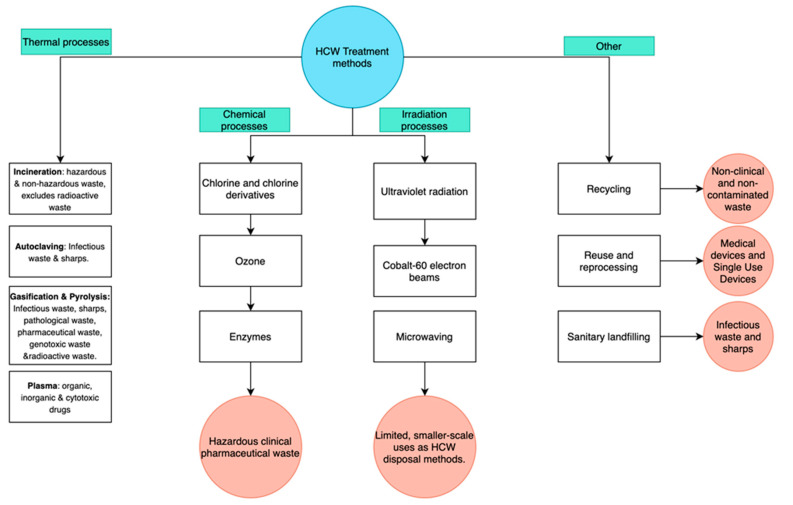
Categories of HCW methods.

The currently used methods of HCW treatment and disposal can be broken down into three main categories (Figure 2): treatments involving 1. Thermal processes, 2. Chemical processes and 3. Irradiation processes [59]. Other methods of HCW treatment that do not include any of the three categories are: landfilling, safe reuse after reprocessing and recycling. The method/process employed depends on numerous factors including: the type of HCW, the equipment and facilities available, operations and maintenance availability, physical space available skillset of employees, regulatory requirements, public acceptability, costing, volume and type of waste [13] The following section outlines the various uses, limitations and strengths of each of the methods. However, specific mechanisms of operation of the methods are not the focus on this review.

### 4.1. Thermal Processes

#### 4.1.1. Incineration

Incineration is one of the most widely used disposal methods for HCW [45]. It involves using a high temperature (800–1100 degrees Celsius) and a dry oxidation process. HCW incineration can employ one of three basic incinerator types: 1. Double-chamber incinerators which are often designed for infectious HCW; 2. Single chamber incinerators, not as widely employed and 3. Rotary Kilns which can be used on genotoxic waste and heat resistant chemicals. Incineration can be broadly applied to HCW as it can be utilised on both hazardous and non-hazardous waste [60] but cannot be applied to pressurised gas containers, silver salts, reactive chemicals, waste high in mercury or cadmium, halogenated plastics or sealed ampoules [13]. The process converts the waste into ash and gases and is often used for various types of pathological waste. While effective, it is more expensive than a method like landfilling by approximately a factor of three to five per unit volume [61]. The method also produces potentially dangerous dioxin emissions which can vary depending on the type of incineration employed and the equipment. Many of these dioxins are classified as known human carcinogens [62]. Strict controls are required to ensure incineration does not exceed the dioxin standards [63]. To mitigate against the release of these harmful pollutants the emissions undergo flue gas treatment. This treatment is not uniformly managed and in many developing countries incinerator malfunctions have led to the potential release of harmful dioxins, furans and antineoplastic [64,65,66]

#### 4.1.2. Autoclaving

Autoclaving is a HCW disposal technique that has been around since the 1800 s as a means of sterilisation [67]. The method utilises moist heat at under pressure to kill microorganisms. Autoclaves can heat up to 250 degrees Celsius, but most autoclaves operate at around 160 degrees Celsius. Autoclaving of clinical waste is viewed as an alternative to incineration but is often much more expensive [68]. Alongside a steeper cost autoclaving is not suitable for chemotherapy treatment waste, volatile or semi-volatile organic compounds, mercury, radioactive waste, hazardous chemical waste or body parts with a large mass [53]. Autoclaves are often seen as a specialised item and many hospitals are not routinely equipped with them [69]. Utilisation of an autoclave also requires that facilities have a drying mechanism and a shredder to reduce waste volume prior to autoclaving. The process also produces a foul odour [70].

#### 4.1.3. Steam Augur

The use of a steam augur involves utilising time and heat to kill microorganisms. This method differs from autoclaving in that it operates at atmospheric pressure and also requires that waste is shredded prior to the process [58].

#### 4.1.4. Gasification and Pyrolysis

Gasification converts solid waste into a combustible gas using a co-reactant and a high temperature of up to 1000 degrees Celsius. These gases can then be integrated into some other form of energy technology [71]. Pyrolysis is similar to gasification, but combustion takes place in the absence of oxygen. Both processes allow for greater volume reduction in waste and are self-sustaining processes [54] but require a high activation energy and the necessary facilities [72]. The processes also require highly specialised, technical personnel to operate them [70]. The requirements for carrying out these methods mean they are not suitable for most standard healthcare facilities.

#### 4.1.5. Plasma

Plasma processing uses an electrical current that is discharged through an inert gas to ionize and in turn causes an electric arc to create high temperatures of up to 1700 degrees Celsius which leads to the decomposition of the compounds [73]. This process van be applied to both organic and inorganic materials and can be applied to highly cytotoxic drugs as well. After this destruction the waste is converted to glass, rock, ferrous metal and inert gas. [48]. While the method looks promising due to its lack of dangerous emissions, it is not currently widely employed in healthcare settings due to its high energy consumption, associated high costs, the requirement of refractory material and the limited lifespan of plasma torch with electrodes [74].

### 4.2. Chemical Processes

A wide variety of chemicals can be used to for chemical disinfection and treatment of HCW. These chemicals include alcohols, acids, alkalis, phenols, halogens, heavy metal compounds, detergents, anti-metabolites, peroxides and enzymes and many produce DBPs (disinfection by-products) [75]. Chemicals such as chlorine and ozone are often used to perform chemical disinfection of hazardous clinical material such as pharmaceuticals that may cause harm and even chromosomal abnormalities to those not intended to be treated [76,77]. DBPs are a known health risk and have been linked to various cancers [78] as well as respiratory irritation, worsened allergies and asthma [79].

### 4.3. Irradiation Processes

#### 4.3.1. Ultraviolet

UV (Ultraviolet) is an electromagnetic wave between length 200 nm and 400 nm. The investment and operational costs of UV are low, it is not highly favoured as method as a method as it lacks penetrative ability and as such is not feasible as a HCW treatment method. It is more commonly employed as one of the procedures in place to treat hospital wastewater [80] alongside other techniques but is usually not applied to HCW in general. It can also be hazardous if protective measures are not taken for the user as exposure can cause in changes to DNA result in various forms of cancer and as such appropriate materials, equipment and training of personnel is required which is not feasible for many healthcare facilities [81].

#### 4.3.2. Cobalt-60 Electron Beams

Cobalt-60 produces gamma rays when it self-disintegrates to act as a disinfectant. The rays of high energy electrons have a deep penetrative ability and are therefore highly effective at killing microorganisms [82,83,84]. While the method is effective to treat waste, it requires highly specialised personnel as gamma irradiation can cause numerous associated health problems to exposed individuals. Such problems include various cancers and even changes in cardiac structure and function [84].

#### 4.3.3. Microwaving

Microwaves are electromagnetic waves with frequencies between radio and infrared waves. In order for microwaving to be used as a process the HCW must be wet. Some treatment processes utilise microwaves to heat water to form steam, which is then applied to the clinical waste stream. Microwaving heats the clinical waste from the inside of the materials to their external surfaces. Microwaving allows for a drastic reduction in waste volume and is environmentally sound [13]. However, microwaving, overall is more costly than larger scale methods such as incineration as microwaving can generally only be carried out on a small scale [53]. Another issue with microwaving as a method of disposal is the potential operational and maintenance issues and expense that may arise [13]. Microwaving also requires the use of noisy shredders and is known to produce foul odours [69]. While microwaving is still a currently utilised method of HCW disposal in both developed and developing countries [85] some studies have indicated it may not provide the level of sterilisation required to completely kill some microorganisms [53,86]

### 4.4. Other Methods of HCW Treatment and Disposal

#### 4.4.1. Recycling

Recycling HCW is often limited to non-clinical waste and non-contaminated waste that poses no threat to either people or the environment and as such does not account for a large portion of generated HCW. This typically includes administration waste such as paper and plastic as well as medical and pharmaceutical packaging including glassware that has been decontaminated [87]. Despite the ability to recycle numerous parts of HCW in hospitals, primary care centres and private clinics there are numerous legal and logistical barriers to recycling in these facilities [88].

#### 4.4.2. Reuse and Reprocessing

Disposable materials and consumables are heavily relied upon to allow for infection control and contagion measures, this, however, generates immense waste and cost. Certain healthcare tools such as reusable trays, surgical gowns, scalpels and sharps containers have a lower environmental and financial cost than their single-use counterparts once sterilised [56]. Alongside consumables, single-use medical devices (SUDs) are used by healthcare providers in place of repairing, cleaning and sterilizing a device that has already been used. Some such items include catheter equipment that can cost up to 4500 euro each. This has led to many healthcare providers reutilising SUD after they have been sent back to the providing company sterilised, reprocessed and sold back to the healthcare provider at a reduced cost to reduce overall costs and waste. While regulated SUD reuse is favourable for the environment many unregulated reuses of SUDs are taking place [89] and without the appropriate legislation and regulation, this may pose a risk to patients using SUDs more than once. However, it has been demonstrated that even after sterilisation for reprocessing harmful pathogenic microorganisms can still be detected [90].

#### 4.4.3. Sanitary Landfilling

Landfilling can be used as the primary method of disposal or for waste that has already been treated with a prior method. As a method of HCW disposal is very common due to its low cost. While landfill is a simple concept, proper management is a necessity, or it can become a public health concern and has been associated with public health issues and has been associated with public health issues such as soil and water contamination both of which can lead to a major public health problem [69]. Dangerous gases including volatile organic compounds and, particularly, benzene, toluene, ethylbenzene and xylene isomers (collectively called BTEX) which can be harmful to human health [91], as well as harmful emissions, landfill leachate is another potentially dangerous side effect of landfilling [92]. Nwachukwu and Anonye, [93] highlight that landfilling of HCW results in GHG emissions [94]. Similarly, Nock et al. [95] suggest that carbon inefficiency of landfill is increased due to the scrubbing required of landfill gas. Moreover, inefficiencies in the landfilling process like mixing of hazardous and non-hazardous wastes at healthcare centers, lack of resources and training of staff further reduces its effectiveness as a disposal method [68]. Despite this, it requires less specialised skills than previously mentioned methods and might be favoured for that reason, particularly in developing countries [16]. Additionally, the EU have implemented landfill directives that have continually required the reduction in biodegradable landfill waste [60]. This constant reduction requirement will lead to less use of landfilling overtime [96].

While the range of methods as described above are used for different categories of HCW, each has limitations that can have detrimental effect of the environment and consequently on global health. It is therefore crucial to make sustainable selection of HCW treatment technologies albeit it being a challenging decision-making issue that requires managing multiple stakeholders and conflicting evaluation criteria [94,97]. Prior research outlines different decision-making models that can facilitate the complex decision-making process of selecting sustainable HCW treatment technologies [98,99]. Similarly, Özkan [100] outlines that the choice of HCW treatment technologies must be based on technical, environmental, economic, and social point of view. These decision making complexities are further amplified in case of developing economies, owing to resources constraints [101,102] and the poor management of HCW results in environmental damage like contamination of ground water by crude medical waste in landfills and health problems such as of spread of diseases by viruses and micro-organisms [103].

## 5. Global Health Consequences of Proper and Improper HCW Disposal

The global health consequences of HCW disposal methods often differ depending on how developed the country is [104]. WHO [2] estimates that 85% of HCW is non-hazardous, despite this, the methods taken to dispose of the waste such as incineration and landfilling can lead to the production of hazardous chemicals and pollutants that can damage the environment and have global health consequences. Pollution is the greatest environmental cause of disease and premature death around the globe and the healthcare sector has been noted for being a significant contributor to acid rain, greenhouse gas emissions, smog, air pollutants, stratospheric ozone depletion and carcinogenic and non-carcinogenic air toxics [105]. Inefficiencies in landfilling process has been noted for producing airborne contaminants such as dioxins and furans that increase the likelihood of cancer, liver failure and various respiratory diseases [69]. There has been a clear link between the increased risk of non-communicable disease and increased exposure to pollution [81,106]. There has also been a growing agreement amongst public health experts that air pollution, even at tolerable levels, aggravates morbidity particularly in respiratory and cardiovascular diseases and leads to premature mortality [107]. Together with the health impact another vital aspect associated with the increasing air pollution is the social costs linked with it [108] highlight the magnitude of social cost of health damages of air pollution and emphasise the urgency to tackle the issue.

Pollutants such as Carbon monoxide, Carbon Dioxide, Nitrogen oxides and Sulfur Dioxide from HCW disposal have led to global warming and as such many related diseases have affected global populations [109,110]. Heat related morbidity and mortality, particularly in developing countries [111] amongst the elderly [112] is a major cause of death [113] Global warming has also led to the emergence of numerous parasitic diseases, often in parts of the world never previously seen [114].

The hazardous HCW that accounts for 15% of medical waste and may be infectious, toxic or radioactive, in the developed world, this once again leads to pollutants in order to dispose of the waste. In the developing world, as well as pollutants from formal disposal methods there is also a greater chance a member or the public may be exposed to this hazardous waste without it having undergone any treatment. A person who experiences one needle stick injury from a needle used on an infected source patient has risks of 30%, 1.8%, and 0.3%, respectively of becoming infected with HBV, HCV and HIV [2]. In many developing countries, hazardous waste such as needles are left at municipal sites, making this a risk [115]. Similarly, anatomical waste has also been improperly disposed of and posed risk of infection [116].

Inappropriate dumping of HCW is often typically assumed to be an issue that predominantly effects the developing world. However, the developed world has less barriers and restrictions from accessing vital prescription medications and as such they are in greater circulation [117]. This too, has led to the inappropriate disposal of medications by civilians [118]. It has been reported that people have disposed of medications they no longer require in their household waste bins which can end up in municipal water supplies [119]. This has led to an increasing concern over antibiotic resistance from antibiotics improperly disposed of [120].

While many countries are working on decreasing their greenhouse gas emissions and finding more novel and “greener” methods for disposing of HCW, this has been a notable challenge during the 2019 coronavirus (COVID-19) pandemic. There has been a mass increase in the use of disposable healthcare products including personal protective equipment this means that even countries such as China, with some of the most sophisticated HCW disposal methods worldwide have been put under immense pressure, operating incinerators at their maximum capacity [121]. The COVID-19 pandemic has led to a huge increase in the employment of single-use plastic which subsequently has led to the utilisation of landfilling and incineration leading to the production of almost 250 tons of waste per day, six times more than their typical average [122,123]. Single use plastics are not the sole concern throughout the pandemic. The increased use of glassware, copper, cardboard, silicon and steel all increased during this time period and were also in need of treatment and disposal [122]. Alongside this, in Wuhan, at the epicentre of the outbreak attempts were made to employ more eco-friendly methods of HCW disposal to aid the huge volumes in need of disposal and treatment. The city utilised previously under-utilised non-incineration methods of disposal. These included: autoclave, dry heat, chemical and microwaving techniques [122]. Despite having some of the most innovative methods of waste disposal globally, China, alongside using greener methods, employment of their incinerators and landfilling at maximum capacity numerous parts of Wuhan still had to employ emergency disposal equipment and methods [122]. The COVID-19 virus has affected some 213 countries globally leading to almost 3 million deaths [124]. Most of these countries are not equipped with the technology, equipment, funding or well-governed legislation to management the HCW disposal as well as China and as such their waste practices will have even greater and far reaching negative impacts on the global environment. Prior to the COVID-19 pandemic, some 2 billion lacked access to any form of waste collection and a further 3 billion had no access to controlled waste disposal [125]. This sudden increase in global pollutant production will undoubtedly have an offset global effect on health in the coming years. Therefore, as the quantities of medical waste generation and consequently requirements for its disposal are increasing, it is imperative to design policies, regulate programs and healthcare waste strategies more methodically and stringently to reduce costs and manage healthcare waste appropriately [69]. To reduce the risks to global health from HCW, advanced techniques need to explored as alternative to current mechanisms and policies designed to facilitate their uptake in a cost effective and strict manner.

### Developing and Under-Developed Countries

While treatment and disposal methods in the developed world are varied and governed by national and international legislation, in the developing world there is a notable lack of appropriate and enforceable law surround HCW treatment and disposal [126]. Developing countries often have to deal with environmental and social problems such as over-crowding and densely packed populations which leads to more waste and by extension more potential for public exposure to HCW. Despite this, developing countries are expanding on the number of healthcare facilities such as laboratories, clinics and hospitals [127]. This increase in healthcare infrastructure has led to an increase in HCW and in most healthcare establishments staff lack the necessary education and training to properly deal with HCW. Similarly, there is a notable lack of facilities, funding and storage for sufficient systems [128]. Of the hospitals that do have facilities and equipment for incineration on site, many are not operational [129]. In 2015 it was found that just over half of healthcare facilities from countries sampled had the necessary systems and equipment for the safe disposal of HCW [130]. All of these factors lead to numerous issues surrounding how HCW is dealt with. Due to the lack of formal training non-clinical waste is often sent for unnecessary internal incineration with hazardous clinical waste leading to over-crowding, overuse and over-whelming of staff and equipment carrying out incineration [131]. Equally, HCW is disposed of alongside domestic waste and ends up at local municipal government waste sites [132,133]. As a consequence of this, there is endemic disease amongst waste cleaners, recycling waste operators, waste pickers and collectors [134]. Open dump burning of potentially dangerous HCW is also an issue [67]. Some hospitals have reported disposing of their HCW to the maximum capacity and disposing of the rest at municipal dumps [116] and some of the HCW is recovered and later sold [135]. One of the major contributors to inappropriate dumping of HCW is a lack of knowledge, training and awareness of healthcare staff within their various centres [136,137]. The lack of clear knowledge and practices has led to toxic and sometimes hazardous waste exposed to the general public. In a study of healthcare centres and hospitals in the Gaza strip, Caniato et al. reported almost 75% of hazardous waste was left untreated and even anatomic waste improperly disposed of and available for scavengers [91]. While this might seem like a problem throughout local centres in the developing world this is often a bigger, more national issue with a lack of clear policies and legislation from government bodies [35,138,139].

## 6. Discussion and Conclusions

While new and innovative technological solutions to waste treatment are continuously being developed, many have no applicability on a large scale in the healthcare settings that need them most. Most solutions that address waste disposal and treatment problems focus on internal or local initiatives and road maps run by a hospital or group of hospitals [139,140]. Although participation in such initiatives aim to make a small but notable contribution, many settings lack basic knowledge of HCW solutions and do not even practice basic waste segregation [141]. While there is no shortage of methods (Figure 2) that can be employed to treat or dispose of waste, it is the acquisition of such equipment, necessary education and training and basic knowledge of waste segregation that seem to be lacking, particularly in developing countries where such resources are scarce. Similarly, many methods, such as incineration, when used correctly are deemed safe, but proper adherence to guidelines and legislation is vital to ensure such safety and strict adherence is not always observed.

While lots of developed countries are making smaller, conscious changes such as improved decision-making tools for resource utilisation, better, more appropriate utilisation of current resources and participation in improvement initiatives such as recycling, reuse and reprocessing methods initiatives there is requirement for these to be encouraged and employed on a greater scale.

Improved technological changes are often small and it can be difficult to roll out a huge technological improvement across the world. It is also noteworthy that even if such education is provided and global understanding of HCW disposal improves, we are still left with many issues surrounding HCW disposal that remain unsolved such as pollution, global warming and the offset effects on global health. There is a real need for a mass overhaul and change in utilization of methods undertaken in HCW disposal especially in the developing world. Open pit burning of hazardous and non-hazardous waste alike remains an issue in many parts of the world. This lack of any prior inertisation means huge volumes of toxic, noxious gases are released into the atmosphere and affect public health on a global scale. Additionally, there is still a huge gap of knowledge between the developed and developing world when it comes to HCW treatment and disposal, however levelling this playing field does not solve the problems associated with HCW disposal. The current scalable, usable and realistic methods of HCW disposal come with a wide array of problems, primarily due to lack of adequate resources and non-adherence to guidelines and legislations, many of which cause lasting damage to the environment and consequently to global human health. While there are initiatives and programs to lessen the volume of waste in need of disposal and also educational programs to better equip certain parts of the world with improved practices, these improvements are only few and far between. As a result, even the most sophisticated solutions to global waste, on a large-scale cause lasting damage to our environment and our health. Additionally, while there is often public attention drawn to reduction in waste on a household and commercial level, there is rarely specific attention drawn to HCW. This is a problem considering the notable contribution HCW makes to global waste. Many studies suggest possible solutions to HCW management problems as identified above but only few implementation, feasibility and follow-up studies. Similarly, many studies draw parallels between the contribution of HCW to greenhouse gas emissions, but few directly link certain facilities with an onset or worsening of a chronic disease. Studies like this are often done for infectious diseases in the developing world and to a lesser extent in developed countries. Further, more in-depth, detailed studies should be carried out to better understand the specific risks posed by numerous HCW methods and the diseases they have directly affected.

This review provides an overarching view of the many HCW methods used and how they play a role in HCW management. It contributes to the body of knowledge by examining not only newer, more experimental HCW methods but also assessing why currently employed methods are still limited in their effectiveness. Additionally, highlighting how global health consequences of HCW disposal methods differ in developing and developed country. However, this study has limitation, the review does not discuss, in detail, the specific mechanisms of operation of each of the methods employed. Moreover, with a notable lack in the literature of specific data on the direct, explicit effects that HCW has on human health the review is limited in its examination of the topic and drawing detailed connections between the negative effects HCW has on health. This review, therefore, demonstrates the need for more in-depth, exhaustive studies on this topic as we have presented the preliminary findings that suggest modern HCW waste practices are inadequate and have adverse effects on health.

## Figures and Tables

**Table 1:** HCW generated (kg/bed/day) per country.

Region	Country	HCW Generated (kg/bed/day)	References
Europe	Ireland	7.7	[15]
	UK	3.3	[16]
	Bulgaria	2	[16]
	Italy	4	[17]
	France	3.3	[16]
	Germany	3.6	[18]
	Greece	3.6	[19]
	Netherlands	1.7	[18]
	Norway	3.9	[16]
	Spain	4.4	[16]
	Latvia	1.18	[20]
America	USA	8.4	[16]
	Canada	8.2	[21]
	Argentina	3	[22]
	Brazil	2.94	[21]
	Ecuador	2.09	[23]
	El Salvador	1.85	[24]
Asia	Bangladesh	1.24	[25]
	China	4.03	[26]
	India	1.55	[25]
	Indonesia	0.75	[27]
	Iran	3.04	[25]
	Japan	2.15	[17,28]
	Jordan	2.69	[17]
	Korea	2.4	[29]
	Laos	0.51	[30]
	Malaysia	1.9	[25]
	Pakistan	2.07	[16]
	Palestine	2.02	[31]
	Thailand	2.05	[32]
	Turkey	4.55	[17]
	Nepal	0.5	[25]
	Lebanon	5.7	[33]
	Kazakhstan	5.34	[20]
	Vietnam	1.57	[25]
Africa	Algeria	0.96	[34]
	Cameroon	0.55	[35]
	Egypt	1.03	[36]
	Ethiopia	1.1	[37]
	Mauritius	0.44	[28]
	Morocco	0.53	[38]
	Sudan	0.87	[39]
	Tanzania	0.75	[17]

Source: Adapted and updated from [40].

## Data Availability

No new data were created or analyzed in this study. Data sharing is not applicable to this article.
